# Adherence to combined Antiretroviral therapy (cART) among people living with HIV/AIDS in a Tertiary Hospital in Ilorin, Nigeria

**DOI:** 10.11604/pamj.2019.32.10.7508

**Published:** 2019-01-07

**Authors:** Chukwuma Anyaike, Oladele Ademola Atoyebi, Omotoso Ibrahim Musa, Oladimeji Akeem Bolarinwa, Kabir Adekunle Durowade, Adeniyi Ogundiran, Oluwole Adeyemi Babatunde

**Affiliations:** 1 National AIDS/STIs Control Programme, Federal Ministry of Health, Abuja, Nigeria; 2 Department of Epidemiology and Community Health, University of Ilorin Teaching Hospital, Ilorin, Nigeria; 3 University of British Columbia, 2329 West Mall, Vancouver, BC, Canada; 4 Department of Community Medicine, Federal Teaching Hospital, PMB 201, Ido-Ekiti, Nigeria; 5 Department of Community Medicine, Afe Babalola University, Ado-Ekiti, Nigeria; 6 World Health Organization Country Office, Lagos, Nigeria; 7 University of South Carolina, Columbia, SC, 20208, USA

**Keywords:** Adherence, antiretroviral, Human Immunodeficiency Virus, Nigeria

## Abstract

**Introduction:**

This study aims to assess the treatment adherence rate among People Living With HIV/AIDS (PLWHA) receiving treatment in a Nigerian tertiary Hospital.

**Methods:**

This was a cross-sectional study that assessed self-reported treatment adherence among adults aged 18 years and above who were accessing drugs for the treatment of HIV. Systematic random sampling method was used to select 550 participants and data were collected by structured interviewer administered questionnaire.

**Results:**

The mean age of respondents was 39.9±10 years. Adherence rate for HIV patients was 92.6%. Factors affecting adherence include lack of money for transportation to the hospital (75%), traveling (68.8%), forgetting (66.7%), avoiding side effects (66.7%), and avoiding being seen (63.6%).

**Conclusion:**

The adherence rate was less than optimal despite advancements in treatment programmes. Adherence monitoring plans such as home visit and care should be sustained.

## Introduction

Human Immunodeficiency Virus (HIV) infection remains one of the ten leading causes of death in Nigeria [1] and the social stigma associated with this disease compounds the problem. Recent estimates suggest that Africa has about 1.7 million of the global 2.5 million individuals newly infected in 2007 [2]. Antiretroviral therapy is indicated for all patients with obvious AIDS defining illness (WHO stage 4) and those with CD4 count less than 200/mm [3, 4]. However, the current view is that cART should be initiated when the CD4 count is 350/mm^3^ for effective care [4]. Adherence is the extent to which a person's behavior in taking medications corresponds with the agreed recommendation from a health care provider [5]. Rates of adherence for individual patients are usually reported as percentage of the prescribed doses of medication actually taken by the patient over a specific period [1, 6, 7]. However, there is no consensual standard for what constitutes adequate adherence. Some trials considered rates greater than 80% to be acceptable whereas others regarded rates of 95% and above to be mandatory for adequate adherence, particularly among patients with serious conditions such as infection with human immunodeficiency virus [8]. Adherence to antiretroviral therapy is an essential component of individual and programmatic success and adherence rates exceeding 95% are necessary in order to maximize the benefit of ART as is crucial for the suppression of viral replication, thus avoiding the development of drug resistance and ensuring the prevention of transmission in discordant couples [1, 9-11]. In Nigeria, an estimated 4.4% of adults between the ages of 15-49 years are living with HIV [12]. The disease has overwhelmed the Nigerian health system, increased the cost of achieving developmental goals by decreasing the size of the workforce and increased the number of orphans and vulnerable children due to AIDS [13, 14]. However, recent studies in Africa have revealed a suboptimal medication adherence of about 77% [9, 15]. In Nigeria, ART treatment adherence rates of 54% and 62.6% have been documented at the Aminu Kano Teaching Hospital Kano [16] and Federal Medical Center, Makurdi respectively [17]. This reported suboptimal treatment adherence to ART will pose serious problems to the control of HIV/AIDS in Nigeria. In patients with HIV, the resistance to antiretroviral drugs has been linked to lower levels of adherence [18, 19]. Partial or poor adherence at levels markedly less than 95 percent can lead to the resumption of rapid replication, mutation to treatment resistant strains of HIV and reduced survival rate of the patients [19].

Stigmatization has been documented as one of many factors related to varying ART adherence [20]. Between 3.5% and 14.6% of women in Africa have reported experiencing a violent reaction from a partner after HIV disclosure [20, 21]. Reports have also revealed the extents to which people are stigmatized and discriminated against by the health workers and the health system [4, 22, 23]. Stigmatization as a factor against optimal treatment adherence has been documented severally [24-27]. Also, the regular availability of the antiretroviral drugs and their accessibility are important factors that affect adherence. The antiretroviral drugs are very expensive, although their cost has been subsidized in the developing countries by the governments and International Organizations. Moreover, stock out of drugs has been identified as a barrier to treatment adherence especially in the developing countries [16, 17, 28, 29]. The health system can create barriers to adherence by limiting access to health care using a restricted formulary, switching to different formulary and having prohibitory high cost for drugs [30, 31]. The success of adherence strategy depends on the education of the patients before initiation of therapy, and co-operation between the patient and the health care provider [32-34]. Factors that strengthen the relationship between the patient and provider include perception of provider's competence, quality and clarity of communication, compassion and involving the patient as an active participant in the treatment decision of the regimen [20]. Having a trusting relationship with a health care provider has also been reported as a facilitator of adherence [32, 35, 36]. Possible side effects of drugs, patient's forgetfulness, depression, being too busy and being away from home have been identified as barriers to adherence [28, 37]. Also, active substances have been documented as one of the strong predictors of non-adherence [38, 39]. Nevertheless, even active substance abusers can have good adherence if the provider takes time to address the patient's concerns about medication, including anticipation of and management of side effects [40]. In the developing countries, financial constraints hampers transportation to the health facility and purchase of food have been found to be great barriers to cART adherence [27, 41, 42]. Other barriers affecting drug adherence include fear of disclosure, trouble incorporating work and family responsibilities with cART, travelling long distances to receive treatment and complicated regimen [30, 40, 41]. With the ART medication adherence in Africa estimated at 77% [15] and Nigeria being the 3rd in the burden of HIV infection in Sub-Saharan Africa [4], there is a challenge in ensuring an optimal level of treatment adherence to prevent the development and spread of resistant strain of the Human Immunodeficiency Virus. Effective treatment is the only option to prevent this complication and prolong life; which can only be achieved with optimal treatment adherence rate of 95% and above, required for the effective suppression of viral replication and prevention of drug resistance [9]. Optimal management of PLWHA can be ensured if clients with indicators of poor adherence can be identified and closely monitored. The objective of this study was to assess the treatment adherence rate and the factors affecting adherence among PLWHA.

## Methods

This study was conducted at the HIV clinic of University of Ilorin Teaching Hospital (UITH), Kwara State, a tertiary health care centre in middle-belt, Nigeria in 2012. It was a descriptive survey with analysis of the observed variables in PLWHA aged 18 years and above who are accessing treatment at the clinic. Self-reported treatment adherence rate was assessed among the respondents over the preceding 7 days to minimize recall bias. Respondents were asked to indicate how many pills they missed during each of the previous 7 days. The mean percent adherence was calculated based on the total number of pills taken divided by the total number of pills respondents reported being expected to take or prescribed by the doctor. In this study treatment adherence was defined as at least 95% of the prescribed dose of cART taken by the patient [8]. Non-adherence to treatment was those patients who score below 95%. Consenting PLWHA who have been on antiretroviral therapy for at least 3 months and not on admission were included in the study while PLWHA with co-morbidities such as tuberculosis, Diabetes Mellitus, Hypertension, mental disorders, hepatitis or any other medical conditions whose medications may interact with the antiretroviral drugs were excluded. The minimum sample size of 550 was determined using the Fishers' formula

$${\text{n}=\text{z}}^{2}{\text{pq/d}}^{2}$$

where: z^2^ is the abscissa of the normal curve that cuts off an area α at the tails (the value for z is found in statistical tables which contain the area under the normal curve and z = 1.96 for 95 % level of confidence); p is the estimated proportion of an attribute that is present in the population (proportion of patients that never returned for further visits after the first clinic visit when cARTs were dispensed in Nigeria was put at 43.4% [43]); q is 1-p (56.6%); and d is the acceptable margin of error for proportion being estimated (0.5). Therefore, sample size

$${\text{n}=\text{z}}^{2}{\text{pq/d}}^{2}$$

= 1.962 X 4.3 X 5.7/0.52 = 3.842x24.51/0.25 =94.167/0.25 =377 Due to the high rate of attrition in HIV/AIDS programmes and research, we have increased our calculated sample size by 45%, yielding a total of 550 The HIV clinic of the UITH had about 2200 adults in its record who were accessing antiretroviral drugs. The survey was carried out during the clinic visits and systematic random sampling method was used to select the sample of 550 participants. Data were collected by a modification of adherence survey instrument developed by the AIDS Clinical Trial (ACTG Adherence Instrument) [40]. Data were analysed using the Statistical Package for Social Sciences (SPSS) version 16. The observed similarities and differences were tested using the Chi-Square Test while cross tabulation of necessary factors with observed variables were done. P-value of less than 0.05 was considered significant at 95% confidence level. Informed consent was sought from selected respondents after careful explanation of the purpose of the study and intended respect for confidentiality had been stated. Ethical approval was obtained from the Research and Ethical Committee of the University of Ilorin Teaching Hospital Ilorin, Nigeria. Limitations of the study include the problem of recall bias on the number and periods of missed doses.

## Results

A total number of 550 HIV patients who met the criteria for the study were surveyed. The age distribution of the respondents ranged from 18 to 65 years with the mean age of 39.9 years±10.0 (Table 1). A high proportion (68.7%) of the respondents were below the age of 45 years and 59.8% were females. Also, 67.8% of respondents were married, 13.1% divorced or separated while 12.0% were single. The highest proportions of respondents have formal education (86.7%), were Muslims (49.3%), were self-employed (46.4%), earned less than Twenty Thousand naira monthly (65.5%) and had secondary education as highest level of education (35.8%). Many (37.5%) of the respondents have been on treatment between 1-2 years with mean duration of 1.2 years, (SD=0.5) and most (99.8%) received pretreatment adherence instruction. More than three quarter (86.9%) of the patients did not miss their drugs in the last 3 months while majority (89.8%) of the patients did not miss their drugs in the last 7 days (Table 2). However, there was a drop on the acceptance of missing their drugs (3.1%) when compared with the responses 3 months ago. Adherence rate for HIV patients is 92.6% (Table 3). Close to half (44.4%) of the respondents missed their drugs because they travelled outside their station, 25% because they felt sick and depressed, 33.3% because they ran out of drugs at home, 5.6 % because they felt better and discontinued treatment, 50% due to lack of money for transportation, 37.5% because they wanted to avoid side effects of the drugs, 41.7% simply forgot to take their drugs, 30.5% because they did not want to be seen in the clinic while collecting drugs and 19.8% because they could not refill their drugs in the clinic because of unscheduled public holidays by the government (Table 4). The respondents with no formal education (21.9%) were the highest proportion that missed their drugs. This was closely followed by those with primary education (12.2%) and secondary education (8.1%) respectively. Those with tertiary education were the least proportion (4.5%) that missed their drugs. There was statistically significant association between the level of education and missing of drugs (Not shown in tables). There was statistically significant association between support from friends and family and treatment adherence among patients. About three quarters (68.8%) of the patients missed their drugs because they travelled outside town and there was statistically significant association between travelling outside home and treatment adherence among patients (Table 5). Among the patients that lack money for transportation, 75% of them missed their drugs. Lack of money for transportation was statistically associated with treatment adherence among the patients. Above half (66.7%) of the patients that wanted to avoid side effects of drugs missed their drugs. There was statistically significant association between avoiding side effects of drugs and treatment adherence. A higher proportion (66.7%) of the patients that was forgetful missed their drug. Also 25% of the patients who felt better and needed to discontinue their drugs missed their medication. However, there was no statistically significant association between feeling better and need not continue treatment and missing of drugs. The proportion of patients who missed drug because they felt better and needed to discontinue treatment was 25% while 78.9% of those that feel sick and depressed missed their drugs. There was statistically significant association between feeling sick and depressed and missing of drugs. Three quarters of the patients that ran out of drug at home missed their drugs and there was statistical significant association between running out of drugs at home and missing drugs. Above half (63.6%) of the patients that wanted to avoid being seen in the clinic missed their drugs. There was statistically significant association between avoiding being seen in the clinic and treatment adherence.

**Table 1:** Sociodemographic characteristics of respondents

Characteristic		Frequency (%) n=550
Age group (years)	<25	16 (2.9)
	25-34	157 (28.5)
	35-44	205 (37.3)
	45-54	128 (23.3)
	55-64	30 (5.5)
	65+	14 (2.4)
Mean age (years)		39.9 + 10.00
Sex	Male	221 (40.2)
	Female	329 (59.8)
Ethnicity	Hausa	65 (11.8)
	Yoruba	409 (74.4)
	Igbo	61 (11.1)
	Others	15 (2.7)
Religion	Christianity	266 (48.4)
	Islam	271 (49.3)
	Traditional	13 (2.7)
Marital status	Married	373 (67.8)
	Single	66 (12.0)
	Divorced/Separated	39 (7.1)
	Widow/Widower	72 (13.1)
Education	No formal	73 (13.3)
	Primary	148 (26.9)
	Secondary	197 (35.8)
	Tertiary	132 (24.0)
Employment	Government	116 (21.1)
	Private	150 (27.3)
	Self-employed	224 (46.2)
	Others	60 (18.3)
Monthly income (Naira)	<20,000	360 (65.5)
	>20,000	190 (34.5)
Average daily income ($)	<1	353 (64.2)
	>1	197 (35.8)

**Table 2:** Treatment adherence among respondents

Adherence	Frequency (%)	
	Yes	No
Ever missed taking your drug in the last 3 months	72 (13.1)	478 (86.9)
Ever missed taking your drugs in the last 7 days	56 (10.2)	494 (89.8)

**Table 3:** Treatment adherence rate among HIV patients based on 7 days recall

Total number of pills expected to take in 7 days (a)	Number of pills missed in 7 days (b)	Number of pills taken in 7 days (c=a-b)	Adherence (d=c/a)	HIV patient (%) n=550	Summed adherence (d*n)
14	14	0	0	25 (4.5)	0
14	8	6	42.8	18 (3.3)	771.3
14	7	7	50.0	6 (1.1)	300
14	6	8	57.1	4 (0.7)	228.6
14	4	10	71.4	2 (0.4)	142.8
14	2	12	85.7	1 (0.2)	85.7
14	0	14	100.0	494 (89.8)	49400.0
			Total	550 (100)	50930.0
		Adherence rate	=	(d*n)/n	
			=	50930/550	
			=	92.6%	

**Table 4:** Distribution of the factors affecting adherence among the respondents

Factors	Respondents (%) n=72	
	Yes	No
Travelled outside town	32 (44.4)	40 (55.6)
Felt sick & depressed	18 (25.0)	54 (75.0)
Ran out of drugs at home	24 (33.3)	48 (66.7)
Felt better and need not continue medication	4 (5.6)	68 (94.4)
Lack of money for transportation	36 (50.0)	36 (50.0)
Wants to avoid side effects	27 (37.5)	45 (62.5)
Forgot	30 (41.7)	42 (58.3)
Does not want to be seen in the clinic	22 (30.5)	50 (69.5)
Public holidays	15 (19.8)	57 (79.2)

**Table 5:** Relationship between the factors affecting adherence and treatment adherence among respondents

Factors	Options	Missed drugs in the last 7 days		
		Yes [n (%)]	No [n (%)]	p-value
Travelled outside town	Yes	22 (68.8)	10 (31.2)	<0.01
	No	34 (6.6)	484 (93.4)	
Lack of transportation money	Yes	27 (75.0)	9 (25.0)	<0.01
	No	29 (5.6)	485 (94.4)	
Wants to avoid side effects	Yes	18 (66.7)	9 (33.3)	<0.01
	No	38 (7.3)	485 (94.4)	
Forgot	Yes	20 (66.7)	10 (33.3)	<0.01
	No	36 (6.9)	484 (93.1)	
Felt better & need not continue drug	Yes	1 (25.0)	3 (75.0)	<0.01
	No	55 (10.1)	49 (89.9)	
Feeling sick & depressed	Yes	15 (78.9)	4 (21.1)	<0.01
	No	41 (7.7)	490 (92.3)	
Ran out of drugs at home	Yes	18 (75.0)	6 (25.0)	<0.01
	No	38 (7.2)	488 (92.8)	
Does not want to be seen in the clinic	Yes	14 (63.6)	8 (36.4)	<0.01
	No	42 (8.0)	486 (92.0)	
Lack of support from family/friends	Yes	53 (9.8)	486 (80.2)	0.01
	No	3 (27.0)	8 (73.0)	

## Discussion

This study found out that 89.8% of the patients did not miss their drugs. Of those who missed their drugs only 27.3% of the prescribed antiretroviral drugs were taken by them. The mean adherence rate of all the respondents was 92.6%. Although this was below the optimal level of greater than 95% [1, 8, 9-11], there was an improvement in the treatment adherence level of 70.8% reported by Salami *et al* [44] in the same hospital about ten years ago. This improvement could be associated with the increased access to antiretroviral drugs in the hospital, regular training of the treatment adherence counselors and the shift from multiple pills therapy to combined pills therapy. However, there was an observed variation in favour of improved adherence levels in respondents when compared with their responses if they missed taking any drugs three months ago. This variation could be explained as recall bias on the part of the respondents. It could also be that the respondents wanted to impress the doctor by their responses. This has been demonstrated in many studies concerning therapy for other diseases where patients often say what they think the doctor would want to hear [45]. This study also found that level of education was significantly associated with treatment adherence among the patients. Those with no formal education were the highest proportion (21.9%) that missed their drugs compared to 4.5% of those with tertiary education. This was consistent with the finding by Christensen *et al.*[46] in which they found that patients with higher educational level have higher treatment adherence because they could have better knowledge of the disease and therapy. Employment status was not found to be significantly associated treatment adherence among the patients. However, the unemployed respondents were found to have higher treatment adherence than the employed. Among the patients, the unemployed were the least proportion (5.3%) that missed their drugs compared to 12.9% of the self-employed and privately employed patients (10%) respectively. There was no statistically significant association between level of income and treatment adherence among the patients. This study found out that treatment adherence was higher with shorter duration of therapy. Among the patients, a higher proportion (11.1%) of those that missed their drugs was from the patients that have been on treatment for more than one year compared to 8.5% of those who have been on treatment for less than one year. Longer duration of therapy was not significantly associated with treatment adherence among patients, (p=0.5365). Disclosure of illness to friends and family was found to be significantly associated with treatment adherence. Higher proportion (26.7%) of those that missed their drugs did not disclose their illness to their friends and families compared to 9.7% that did. This finding was consistent with the findings by Mills *et al.* [47] and Chesney *et al.* [20] in which they identified that not disclosing of illness to friends and families could affect treatment adherence. Disclosure of illness to friends and families will help reduce stigmatization and improve support from friends and families.

This study found that support from friend and family could improve treatment adherence. Higher proportion (27%) of patients that missed their drugs did not have support from friends and family. Support from friends and families could reduce psychological stress and financial burden especially in our environment where extended family system is practiced. Lack of money for transportation was also identified as a significant factor affecting treatment adherence. Seventy five percent of the patients who did not have money for transportation to keep hospital appointment missed their drugs. Lack of money for transportation has been reported in a similar study at Enugu, Nigeria by Uzochukwu *et al.* [29] and in Malawi by Yu *et al.* [48]. Possible reasons could be because above half of the patients live below one Dollar per day. Also, it could be that some of the patients travel a long distance outside where they live to access their drugs to avoid being seen by those who may know them. Patients avoiding side effects of drugs was significantly associated with treatment adherence among the patients. Two-thirds (66.7%) of patients who wanted to avoid side effects of the antiretroviral drugs missed their drugs. This finding is consistent with result of a similar studies by Uzochukwu *et al.* [30] in a tertiary hospital in Enugu, Nigeria and among HIV patients by Chesney *et al.* [38] Forgetfulness was found to be significantly associated with treatment adherence among HIV patients. Above half (66.7%) of the patients that were always forgetful missed their drugs. Forgetfulness as a barrier to treatment adherence have also been documented from a study by Ilyasu *et al.* [16] at the Aminu Kano Teaching Hospital Kano, Nigeria and also by Olowokere *et al* [28] among HIV patients in Ibadan, Nigeria. The reason for forgetfulness could be that they were getting better or that they have busy schedules. Running out of drugs at home was significantly associated with treatment adherence. This finding is consistent with the result of the study by Weiser *et al.* [41] in Botswana. Probable reason for running out of drugs at home could be not keeping clinic appointment for follow-up care and drug refill. Patients avoiding being seen at the clinic was also found to be a significant barrier to treatment adherence among the patients. The treatment adherence rate of the patients was 92.6% and there was an improvement in the treatment levels when compared with previous studies. Although this commends the efforts of the government and the development partners, more needs to be done to attain the optimal treatment adherence rate of at least 95%. The current study has some limitations. Being a descriptive approach to determine associations between factors affecting adherence to cART and the adherence rate, causality could coud not be established. In addition, there could be confounding factors that are difficult to ascertain usin a descriptive approach. For example, other comorbidities unrelated to HIV could affect patients' health such that they become sicker and skip their cART on certain days. The self-reporting of the amount of drug taken in the 7 days preceding the data collection is also not free from bias. A person living with HIV/AIDS might have been poor in adhering to treatment in the past month and give a false or misleading account on adherence to please the researchers. These shortcomings notwithstanding, this research has shown the significant associations between the factors affecting adherence and adherence rate. This information about the associations can help guide future research on adherence to cART.

## Conclusion

In conclusion, we were able to demonstrate a mean adherence rate of 92.6% among all the respondents which was below the optimal level of greater than 95% but higher than the treatment adherence level of 70.8% reported [44] in the same hospital about ten years ago. This study has been able to also identify some factors affecting adherence such as lack of money for transportation, forgetfulness, frequent travelling outside home, feeling sick and depressed, running out of drugs at home, wanting to avoid side effects of drugs, and no access to drugs due to unscheduled public holidays. The establishment of initiatives by the government, non-governmental organizations or the communities to address these problems would help patients to keep clinic appointments. A system of financial enhancement to defray transportation cost for every clinic visit could be considered and patients should be given a week appointment before the exhaustion of the prescribed drugs. This could address the problem of running out of drugs during unscheduled public holidays. The findings of this study could also inform other future studies to determine the causal factors related to adherence. Knowing the causes of poor adherence would help to design more targeted and effective solutions.

### What is known about this topic

- Optimal management of PLWHA can be ensured if clients with indicators of poor adherence can be identified and closely monitored;
- ART treatment adherence rates of between 54% and 62.6% have been documented in Northern Nigeria;
- Stigmatization has been identified as one of several factors related to varying ART adherence.

### What this study adds

- This study also found that factors affecting adherence include lack of money for transportation, feeling sick and depressed, running out of drugs at home, wanting to avoid side effects of drugs and no access to drugs due to unscheduled public holidays;
- Support from friends and family was found to be significantly associated with treatment adherence.

## Competing interests

The authors declare competing interests.
